# Disparities in Utilization of Social Determinants of Health Referrals Among Children in Immigrant Families

**DOI:** 10.3389/fped.2018.00207

**Published:** 2018-07-24

**Authors:** Omolara T. Uwemedimo, Hanna May

**Affiliations:** ^1^Department of Pediatrics, Donald and Barbara Zucker School of Medicine at Hofstra/Northwell, Hofstra University, Hempstead, NY, United States; ^2^Department of Occupational Medicine, Epidemiology and Prevention, Donald and Barbara Zucker School of Medicine at Hofstra/Northwell, Hofstra University, Hempstead, NY, United States; ^3^GLOhBAL (Global Learning Optimizing Health Building Alliances Locally) at Cohen Children's Medical Center of New York, New Hyde Park, NY, United States; ^4^Wellesley College, Wellesley, MA, United States

**Keywords:** social determinants of health, immigrant, referral, screening, limited English proficiency

## Abstract

**Objective:** Children in immigrant families (CIF) are at elevated risk of experiencing adverse social determinants of health (SDH), particularly material hardship, which contribute to disparate health outcomes. Previous studies have found that SDH screening programs integrated into pediatric practices have increased receipt of social service resources. Few studies have examined use of social services in these programs among ethnically-diverse patient populations and associations with caregiver immigrant status or limited English proficiency (LEP).

**Methods:** Caregivers of children (<18 years) were routinely screened in a practice-based, SDH screening program offering referral, assisted navigation and follow-up support. Information on caregiver race/ethnicity, US nativity, citizenship status and self-reported English proficiency was collected. Associations with utilization of referral resources at 12 weeks were measured using Chi-square and Fisher's Exact tests.

**Results:** Of 148 caregivers, most were mothers (83.2%) and non-White (91.9%). Over half were born outside of the U.S (59.7%) and one-third were LEP (33.6%). Approximately one-third (30.9%) successfully utilized program-provided resources at 12-week follow-up. LEP caregivers and undocumented caregivers were more likely to be lost-to-follow-up. However, LEP caregivers who remained in the program utilized resources more than English-proficient caregivers (38.4 vs. 18.4%, *p* = 0.031). Similarly, significantly more non-citizen caregivers utilized referrals compared to US citizens (37.4 vs. 23.1 vs. 0.0%, *p* = 0.043).

**Conclusions:** Families with non-US citizen or LEP caregivers were at highest risk of being lost-to-follow-up, but if engaged, were more likely to utilize resources. These findings indicate the need for larger studies to determine how to prevent loss-to-follow-up among immigrant and LEP caregivers participating in SDH screening programs.

## Introduction

Children in immigrant families (CIF) represent one-quarter of all children living in the US—and is projected to rise to one in three US children by 2050 (1). These children, mostly racial/ethnic minorities, are both foreign-born (1st generation) and US-born with at least one foreign-born parent (2nd generation) (2). Currently, CIF are more likely to experience adverse social determinants of health (SDH), particularly material and economic hardship, than children in non-immigrant families (3), contributing to suboptimal health service utilization patterns and increased rates of acute and chronic illness including asthma, otitis media, diarrheal illness, cardiovascular disease, and mental health problems (4–8). Nationally, 39% of all CIF confront difficulties affording food, compared with 27% of children of US-born parents. CIF also are more than twice as likely as children of US-born parents to live in families paying over 50% of their income for housing, and are more than four times as likely to live in crowded housing. Almost one-quarter of all CIF are uninsured, more than twice the rate for children of natives (9). These disparities are partly attributed to the disproportionate rates of parental unemployment and underemployment, limited English proficiency (LEP), and/or restricted access and participation in public safety net programs that exist among immigrant families (10, 11).

In light of the clear health impacts of material and economic hardship, there have been new recommendations for pediatric practices to expand their activities to include identification of unmet social needs and referral to local resources (12). Research from pediatric practices already implementing SDH screening have revealed rates as high as 75% of patients reporting unmet social needs, such as housing deprivation or food insecurity (13, 14). In turn, studies have demonstrated that low-income patient populations respond positively to social needs screening during well-child care, and successfully receive community resources through these systematic screening and referral systems (15–21).

However, immigrant families face unique obstacles within the pediatric medical home that may impair the effectiveness of social need screening programs, including language barriers, mixed eligibility for services, low health literacy, poor patient-provider communication and perception of cultural insensitivity to values and customs (22, 23). For these families, successful utilization of resources may be significantly hampered by difficulty navigating services and barriers to follow-up after referral. This is particularly troubling, since CIF potentially have the greatest benefit from such programs; immigrant families tend to be more socially isolated, limiting their access to information about community resources (24). While previous studies, have examined rates of successful use of services by race/ethnicity, to date, none have examined success rates among children by parental immigrant status and/or parental LEP. Moreover, there is a dearth of research examining successful utilization of services in practice-based, SDH screening/referral programs. Given this knowledge gap in the literature, our study sought to determine the prevalence of successful use of referrals within a pediatric practice providing SDH screening, referral and navigation/ follow-up support. In addition, we sought to examine associations between immigrant family-specific factors, including parental immigrant status and parental LEP, and successful use of services.

## Materials and methods

### Study design

This is a secondary data analysis of results from a pilot program to integrate routine SDH screening aligned with referral, assisted navigation services and follow-up within a hospital-based pediatric practice.

### Study population and setting

This study was conducted in Queens, New York at a large hospital-based pediatric ambulatory practice, with a catchment population that spans all 5 boroughs of New York City and the entire peninsula of Long Island. Patients served by this practice are ethnically and socio-economically diverse with greater than half of patients living in immigrant families from Latin America, East Asia, South Asia, non-Hispanic Caribbean, the Middle East, and Africa; over two-thirds of patients receive health insurance coverage through Medicaid or Medicaid managed care.

The Family Needs Screening Program (FAMNEEDS) was started in August 2016 to identify and address family material and psychosocial needs of patients through: (1) universal screening and assessment of social needs at pediatric preventive care visits for children aged 0–18 years using the FAMNEEDS screening tool; (2) assisted navigation and referral to local community resources by trained student “navigators” and (3) periodic follow-up to ensure successful linkages. This program complements the existing social services support for families, which includes 2 part-time social workers and a full-time child psychologist.

### Data collection

**Figure 1:**
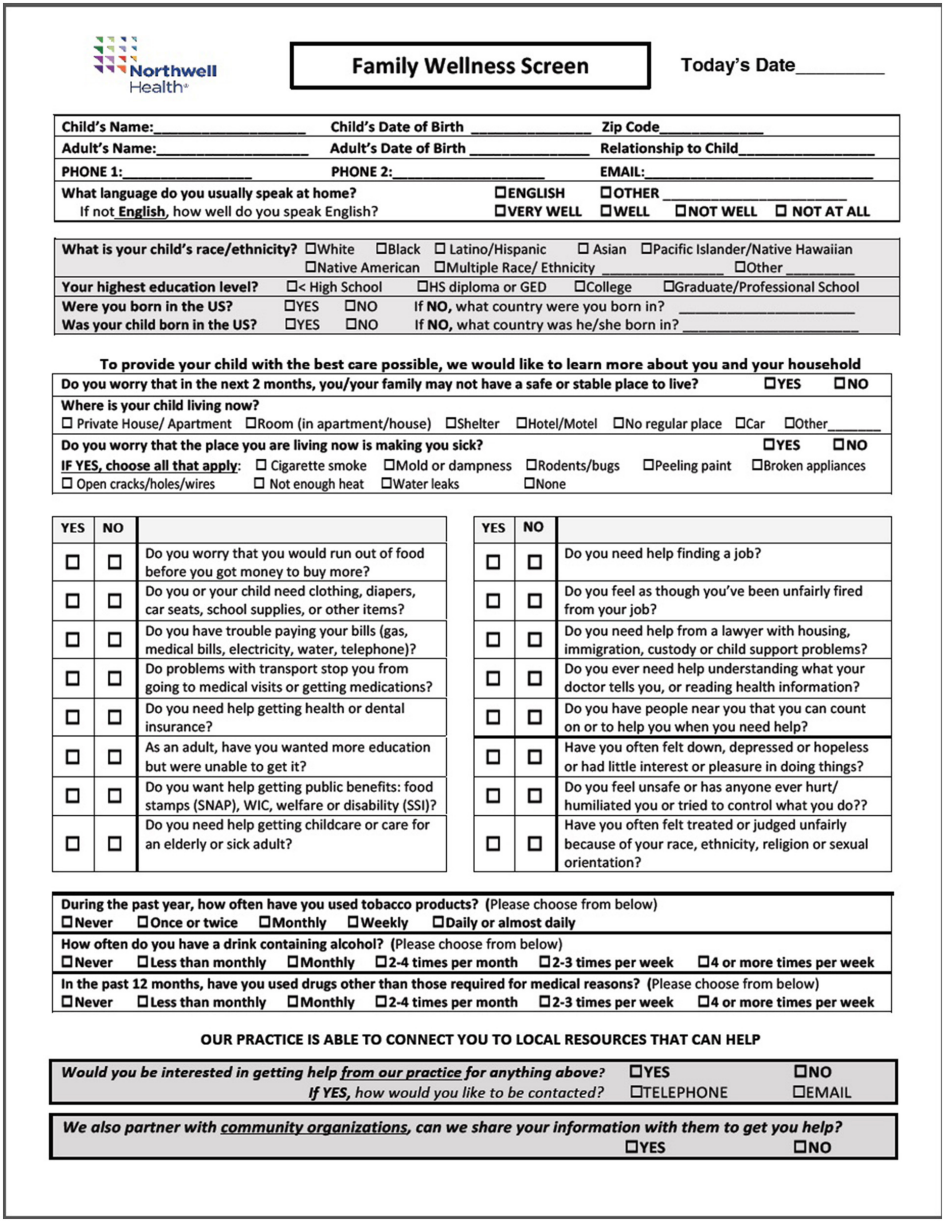
Cohen Children's Medical Center Family Needs Screening Tool.

The FAMNEEDS tool, which has recently been revised (see Figure 1), was designed after rigorous review of multiple previously published tools used for universal SDH screening of families within healthcare practices (12). Prior to study implementation, multiple iterations including different question formats of the tool were tested with caregivers to assess understanding of question constructs, frequency and time duration for completion, particularly among LEP and immigrant families. In addition to assessment of a comprehensive array of social needs (e.g., unemployment, food/energy/housing insecurity, childcare, transport, etc.), the FAMNEEDS tool also captures socio-cultural demographic data, given the diverse patient population. These factors include child/parental nativity, country of origin, parental educational attainment and LEP. The tool was forward and back translated into the 6 most common languages spoken by patient families. The most common languages are Spanish, Haitian Creole, Urdu, Punjabi, Hindi, and Arabic. In order to avoid potential barriers to completion, avoid redundancy with information collected in the electronic health record and maintain brevity, the tool did not capture certain demographic data including insurance type or household income. For caregivers who were illiterate, a navigator was required to administer the screen.

Caregivers accompanying patients aged 0–18 years attending the Cohen's Children's Medical Center General Pediatric Practice (CCMC-GPP) for a preventive care visit were provided with the FAMNEEDS screening tool by a medical assistant after triage, and instructed to complete the screen and give to their provider for review at the start of the visit. Providers were trained to identify positive screens (i.e., a screen with at least 1 social need) among families that desired assistance and informed caregivers that they would receive a follow-up phone call in 48 h from a resource navigator. “Resource navigators” were undergraduate students, trained to navigate online social service databases and/or provide referrals to social service case managers at designated partner community-based organizations. The students' undergraduate field of study included pre-medicine, pre-nursing, sociology, public health, or community health sciences. In order to ensure linguistic and cultural concordance with the ethnically diverse patient population, resource navigators were bilingual in English and at least one of the most common languages spoken by the patient population.

Navigators are rigorously trained in use of 2 comprehensive online social service databases, from which the navigator is able to provide contact information for families with social needs. In addition, the navigator can send direct e-referrals to partner community-based organizations (CBO). Common needs include public benefits such as: Supplemental Nutrition Assistance Program (SNAP), Temporary Assistance for Needy Families (TANF) Special Supplemental Nutrition Program for Women, Infants, and Children (WIC), Social Security Insurance (SSI), Medicaid and other health insurances, legal assistance, immigration assistance, tax assistance, Home Energy Assistance program (HEAP)/ Energy Share applications, childcare, adult education classes, after school programs, and counseling services. When a positive screen with a caregiver requesting help was identified, navigators would conduct an initial “intake” call to obtain detailed information within 48 h and then research potential resources. Identified resources with contact information were provided within 1 week after the intake call. Resource navigators also conducted follow-up phone calls every 2 weeks for a total of 8 weeks to ascertain progress on the referral and/or provide alternative resources, as needed. Lastly, the final “follow-up call” was performed at 3 months post-intake to assess the status of the referral. Three attempts are made for each follow-up call to refer caregivers to appropriate resources or check the status of a previous referral. The Navigator documents all screening information, follow-up call information and outcomes of each call in a HIPAA- compliant, IRB-approved data registry.

### Measures

The main outcome of interest was caregiver self-report of utilization of referral resources at the caregiver 3-month final phone call. This outcome was separated into three categories: successfully used or in progress of using at least one of the referral resources vs. non-utilization of resources vs. lost-to-follow-up (i.e., after referral, parent was unable to be contacted after three attempts). As an additional validity check, we also included a randomly selected subset of 50 caregivers, and compared their self-report to actual use of resources directly through data from a main partner community-based organization, Single Stop. This organization was a direct partner with our program and assisted referred families using a case manager (e.g., completion of enrollment into SNAP, TANF, WIC, SSI, housing and utility programs).

The main predictors assessed included self-reported demographic and socio-cultural information of the child/caregiver dyad. Specifically, these factors included child characteristics such as age, insurance type and caregiver characteristics, such as race/ethnicity, educational attainment, US nativity, citizenship status and LEP. Of note, citizenship status and LEP (using the US Census question “How well do you speak English?”) was determined by self report.

### Analysis

Of the 703 caregivers that were screened, the analytic sample for this study was restricted to families that indicated a need on the screening tool (*n* = 299). Of these families, only those that accepted patient navigator assistance for referrals were included in the analysis (*n* = 148). Descriptive statistics, using univariate frequencies were used to compare the prevalence of successful utilization of resources vs. unsuccessful utilization vs. those that were lost to follow-up among the analytic sample. Chi-square tests were then utilized to determine demographic factors with statistically significant bivariate association (*p*-value ≤ 0.05) with successful/in-process utilization vs. non-utilization of referrals vs. lost-to-follow-up. Lastly, multivariate logistic regression was conducted to control for confounding and identify predictive factors that remained significantly associated with successful utilization. Variables that had >10% missing data were excluded from the final model. SPSS 24.0 was used for all analyses and the study was approved from the Northwell Health Institutional Review Board.

## Results

Of the 148 caregivers in the study, the majority were mothers (83.2%), non-White (91.9%) and graduated HS or obtained a GED (80.6%) (Table 1). Most were U.S. citizens (71.8%) and born outside of the U.S (59.7%). LEP was present in one-third of the sample (33.6%) Approximately one-third (30.9%) successfully utilized program-provided resources at the 12-week follow-up. The percentage of families who did not utilize program-provided resources was lower among caregivers with LEP (18.4 vs. 38.4%, *p* = 0.031). However, LEP caregivers were more likely to be lost-to-follow-up, as well. Immigration status was significantly associated with 12-week follow-up outcome. US citizens were the most likely to not utilize referrals compared to documented and undocumented immigrants (37.4 vs. 23.1 vs. 0.0%, *p* = 0.043).

**Table 1:** Successful utilization of referral (%) stratified by demographic group.

		**Total (*****N*** = **149)**		**Did not use referral (*****N*** = **47)**		**Successfully used referral (*****N*** = **46)**		**Lost to follow-up (*****N*** = **56)**		***P*-value**
**Variable**	**Category**	***n***	***%***	***n***	***%***	***n***	***%***	***n***	***%***	
Relationship to child	Mother	124	83.2	40	32.3	35	31.6	49	39.5	0.146
	Father	19	12.8	6	31.6	7	36.8	6	31.6	
	Grandparent/other	5	3.4	1	20.0	4	80.0	0	0.0	
Immigrant caregiver	Yes	89	59.7	29	32.6	26	29.2	34	38.2	0.504
	No	49	32.9	13	26.5	19	38.8	17	34.7	
Medicaid	No	29	19.5	14	48.3	7	24.1	8	27.0	0.117
	Yes	117	78.5	33	28.2	39	33.3	45	38.5	
Education	<HS	27	18.1	9	33.3	4	14.8	14	51.9	0.335
	HS or GED	32	21.5	10	31.3	11	34.4	11	34.4	
	>HS	88	59.1	28	31.8	30	34.1	30	34.1	
LEP	No	99	66.4	**38**	**38.4**	30	30.3	**31**	**31.3**	**0.031**
	Yes	49	32.9	**8**	**18.4**	16	32.7	**24**	**49.0**	
Social support	No	39	26.2	8	20.5	10	25.6	**21**	**53.8**	**0.042**
	Yes	105	70.5	37	35.2	35	33.3	**33**	**31.4**	
Race	Hispanic	50	33.6	14	28.0	13	26.0	23	46.0	0.369
	White	12	8.1	6	50.0	5	41.7	1	8.3	
	Black	44	29.5	12	27.3	15	34.1	17	38.6	
	Other	38	25.5	13	34.2	11	28.9	14	36.8	
Immigration status	US Citizen	107	71.8	**40**	**37.4**	31	29.0	**36**	**33.6**	**0.043**
	legal permanent resident/temporary protected status/work authorized	26	17.4	**6**	**23.1**	10	38.5	**10**	**38.5**	
	undocumented	11	7.4	**0**	**0.0**	3	27.3	**8**	**72.7**	
Immigration years	US Born	69	46.3	19	27.5	23	33.3	27	39.1	0.291
	</= 3 years	5	3.4	0	0.0	3	60.0	2	40.0	
	4–10 years	23	15.4	6	26.1	6	26.1	11	47.8	
	>10 years	52	34.9	22	42.3	14	26.9	16	30.8	

*Bold values p < 0.05*.

## Discussion

This study represents one of the few studies describing referral utilization patterns by parental immigrant and LEP status in a practice-based, SDH screening program. Among our sample, where over half are children in immigrant families, we found that factors such as immigrant status and LEP were associated with higher rates of successful utilization of resources but also higher rates of lost-to-follow-up. The results from this pilot data suggests two main points within culturally diverse populations: (1) if engaged, children living in households with non-US citizen or LEP caregivers are more likely to utilize resources provided in SDH screening programs offering navigation and follow-up support, but (2) are the most at risk for difficulty in maintaining contact for follow-up support.

Our rate of 31% of families successfully utilizing resources is consistent with other published studies describing practice-based health-related social needs (HRSN) screening programs. Prior work has shown that the integration of navigation services in conjunction with screening and referral has increased effectiveness (17, 25). Controlling for age, race, insurance status, reason for referral, and source of referral, experimental groups who were assisted by patient navigators also had a 39% greater odds of having timely follow-up. Twenty percent of families reporting successful utilization were families referred to our community partner and all of these families did engage with referral resources, as per our community partner.

In contrast, few studies have examined socio-cultural differences in successful resource utilization. The finding of elevated lost-to-follow-up rates among foreign-born parents, particular those with undocumented status, does point to whether cultural differences are contributing to this differential. Qualitative work from Silverstein et.al, identified multiple barriers among ethnic minority and immigrant parents to engagement with community resources for SDHs including: disconcordance with cultural and personal values of self-sufficiency, cultural stigma of help-seeking, fear of governmental intrusion, and negative past experiences that were deemed judgmental or culturally insensitive (22). Interestingly, other immigrant-specific characteristics demonstrated associations with successful use of services, particularly those who were LEP. This may be attributed to the potential benefit of one-to-one navigation services, in contrast to services which solely provide referrals and require patients to navigate resources themselves. Future larger studies are warranted to confirm this increased utilization among LEP participants.

This analysis is to be considered exploratory due to limitations including a small sample size, which lacks the power to detect smaller, clinically relevant differences between groups. We were also unable to utilize regression analyses to control for potential confounding factors. Additional limitations were that our sample included only caregivers who had already accepted navigator assistance for referrals, and utilization of resources, LEP and citizenship were all measured by self-report. The study may be less generalizable as concerted efforts were made to ensure that our program was as culturally sensitive as possible in design and implementation. The screening tool was available in several different languages and bi/multilingual patient navigators were recruited to reflect the needs/accommodate our large, urban immigrant patient population.

Currently, national pediatric recommendations support embedding screening of SDHs into well-child visits and underscore the need for larger studies to determine if SDH screening programs are effective across sub-populations, particularly children in immigrant families. Additionally, qualitative work is warranted to gauge family experiences in these programs, which may help explain the disparate loss-to-follow-up found among undocumented and LEP caregivers. However, if these families are supported with assisted navigation and follow-up, they may have better outcomes than the general pediatric population. The observed differences in utilization outcomes highlight parental English proficiency and nativity as potential demographics to capture in SDH screening tools for consideration when developing SDH screening tools for disadvantaged patient populations.

## Author contributions

OU conducted the study, conceptualized and designed the manuscript, conducted analysis of the interpreted the data and critically revised manuscript. HM conducted analysis of the data, drafted the manuscript, interpreted the data and critically revised the manuscript. All authors approved the final manuscript as submitted and agree to be accountable for all aspects of the work.

### Conflict of interest statement

The authors declare that the research was conducted in the absence of any commercial or financial relationships that could be construed as a potential conflict of interest. The reviewers, PW and RS, and handling Editor declared their shared affiliation.

## References

[1] Seiber E. Covering the remaining uninsured children. Med Care (2014) 52:202–7. 10.1097/mlr.0000000000000039 24309671 PMC3932957

[2] U.S Census Bureau. Summ File (2000) 1:200.

[3] Wight V Thampi K Chau M Poor children by parents' nativity: what do we know? report, New York, NY: National Center for Children in Poverty (2011).

[4] Evans G. The environment of childhood poverty. Am Psychol. (2004) 59:77–92. 10.1037/0003-066x.59.2.77 14992634

[5] Krieger J Higgins D. Housing and health: time again for public health action. Am J Public Health (2002) 92:758–68. 10.2105/ajph.92.5.758 11988443 PMC1447157

[6] Perera F Illman S Kinney P Whyatt RM Kelvin EA Shepard P. The challenge of preventing environmentally related disease in young children: community-based research in New York City. Environ Health Perspect. (2002) 110:197–204. 10.1289/ehp.02110197 11836150 PMC1240736

[7] Cherayil M Oliviera D Sandel M Tohn E Lawyers and doctors partner for healthy housing. Clgh Rev. (2005) 39:65.

[8] Wood D Valdez R Hayashi T Shen A. Health of homeless children and housed, poor children. Pediatrics (1990) 86:858–66. 1701236

[9] ICPSR National Survey Of America's Families (NSAF), 2002. Washington, DC: Urban Institute; Child Trends (2002).

[10] Landale N Thomas K Van Hook J. The living arrangements of children of immigrants. Future Child. (2011) 21:43–70. 10.1353/foc.2011.0003 21465855 PMC3241619

[11] Waters M Jiménez T Assessing immigrant assimilation: new empirical and theoretical challenges. Annu Rev Sociol. (2005) 31:105–25. 10.1146/annurev.soc.29.010202.100026

[12] Chung E Siegel B Garg A Conroy K Gross RS Long DA. Screening for social determinants of health among children and families living in poverty: a guide for clinicians. Curr Probl Pediatr Adolesc Health Care (2016) 46:135–53. 10.1016/j.cppeds.2016.02.004 27101890 PMC6039226

[13] Fleegler E Lieu T Wise P Muret-Wagstaff S. Families' health-related social problems and missed referral opportunities. Pediatrics (2007) 119:e1332–e1341. 10.1542/peds.2006-1505 17545363

[14] Garg A Butz A Dworkin P Lewis R Serwint J. Screening for basic social needs at a medical home for low-income children. Clin Pediatr (Phila). (2008) 48:32–6. 10.1177/0009922808320602 18566347

[15] Garg A Butz A Dworkin P Lewis R Thompson R Serwint J. Improving the management of family psychosocial problems at low-income children's well-child care visits: the WE CARE project. Pediatrics (2007) 120:547–58. 10.1542/peds.2007-0398 17766528

[16] Garg A Sarkar S Marino M Onie R Solomon B. Linking urban families to community resources in the context of pediatric primary care. Patient Educ Couns. (2010) 79:251–4. 10.1016/j.pec.2009.10.011 19962849 PMC2916170

[17] Garg A Toy S Tripodis Y Silverstein M Freeman E. Addressing social determinants of health at well child care visits: a cluster RCT. Pediatrics (2015) 135:e296–e304. 10.1542/peds.2014-2888 25560448 PMC4306802

[18] Gottlieb L Tirozzi K Manchanda R Burns A Sandel M. Moving electronic medical records upstream. Am J Prev Med. (2015) 48:215–8. 10.1016/j.amepre.2014.07.009 25217095

[19] Hassan A Scherer EA Pikcilingis A Krull E McNickles L Marmon G. Improving social determinants of health: effectiveness of a web-based intervention. Am J Prev Med. (2015) 49:822–31. 10.1016/s0749-3797(15)00647-9 26215831

[20] Colvin J Bettenhausen J Anderson-Carpenter K Collie-Akers V Plencner L Krager M. Multiple behavior change intervention to improve detection of unmet social needs and resulting resource referrals. Acad Pediatr. (2016) 16:168–74. 10.1016/j.acap.2015.06.001 26183003 PMC4712125

[21] Peterson EE (2017). Screening Families for Unmet Social Needs in a Pediatric Clinic. Dayton: Wright State University Available online at: https://corescholar.libraries.wright.edu/cgi/viewcontent.cgi?article=1196&context=mph (Accessed January 22, 2018).

[22] Silverstein M Lamberto J DePeau K Grossman D. “You Get What You Get”: unexpected findings about low-income parents' negative experiences with community resources. Pediatrics (2008) 122:e1141–8. 10.1542/peds.2007-3587 19047215 PMC2596660

[23] Coker T Windon A Moreno C Schuster M Chung P. Well-child care clinical practice redesign for young children: a systematic review of strategies and tools. Pediatrics (2013) 131(Suppl. 1):S5–25. 10.1542/peds.2012-1427c 23457149 PMC4258824

[24] Szreter S. Health by association? Social capital, social theory, and the political economy of public health. Int J Epidemiol. (2004) 33:650–67. 10.1093/ije/dyh013 15282219

[25] Fleegler E Bottino C Pikcilingis A Baker B Kistler E Hassan A Referral System Collaboration Between Public Health and Medical Systems: A Population Health Case Report. National Academy of Medicine. 2016.

